# HMGB1-dependent signaling in the regulation of mast cell activity during inflammation

**DOI:** 10.3389/fimmu.2025.1643427

**Published:** 2025-10-03

**Authors:** Justyna Agier, Sylwia Różalska, Magdalena Wiktorska, Elżbieta Kozłowska, Magdalena Jurczak, Monika Nowak, Paulina Żelechowska

**Affiliations:** ^1^ Department of Microbiology, Genetics and Experimental Immunology, Centre of Molecular Studies on Civilization Diseases MOLecoLAB, Medical University of Lodz, Lodz, Poland; ^2^ Department of Industrial Microbiology and Biotechnology, Faculty of Biology and Environmental Protection, University of Lodz, Lodz, Poland; ^3^ Department of Molecular Cell Mechanisms, Faculty of Health Sciences, Medical University of Lodz, Lodz, Poland

**Keywords:** HMGB1, alarmins, inflammation, sterile inflammation, mast cells, pattern recognition receptor

## Abstract

**Background:**

Damaged cells release endogenous molecules known as alarmins into the extracellular space following cellular injury. Alarmins may function as adjuvants by interacting with PRRs to indicate danger and initiate a localized sterile inflammatory response, which facilitates tissue regeneration. A pivotal alarmin is HMGB1, which is internalized through the RAGE to notify adjacent cells about compromised homeostasis. Given the significant role of mast cells (MCs) in inflammatory processes and the critical nature of alarmins as indicators of danger, this study evaluates the hypothesis that MCs serve as essential sensors of cellular injury. The present study investigates whether HMGB1 affects the expression levels of specific PRRs in mature MCs. These receptors include Dectin-1 and Dectin-2, TLR2, NOD1, and RIG-I. Furthermore, this study aims to determine whether HMGB1 modulates the inflammatory response of these cells, which encompasses the production of cytokines, chemokines, ROS, histamine, and cysLTs, as well as their migration patterns. Moreover, the research aims to investigate the role of RAGE and the involvement of signaling molecules in the activation of MCs mediated by HMGB1.

**Methods:**

All experiments were carried out using *in vivo* differentiated, mature tissue MCs freshly isolated from the rat peritoneal cavity. The potency of HMGB1 to provoke MC PRR expression, generation, and/or release of a panel of mediators and migration was investigated.

**Results:**

HMGB1 markedly enhances the expression of Dectin-1, RIG-I, and NOD1, while simultaneously stimulating MCs to produce CCL3, IL-1β, TNF, cysLTs, histamine, and ROS. This protein acts as a potent chemoattractant for MCs. The administration of RAGE antagonist to MCs significantly attenuated the generation of mediators and the migratory response, thereby confirming the receptor’s involvement in the response of HMGB1-treated cells. Intracellular signaling in MCs activated by HMGB1 involves ERK1/2, p38 MAPK, PI3K, NF-κB, and, in part, JAK2.

**Conclusions:**

The data robustly support the notion that HMGB1 is an important endogenous alarmin that promotes and enhances MC activity in inflammatory processes. These insights highlight HMGB1 as a potential therapeutic target for regulating MC-driven inflammatory disorders, which encompass allergy, autoimmune diseases, and chronic conditions.

## 1 Introduction

Recent research has given rise to the concept of “molecular multitasking” biomolecules, which participate in interactions that are not aligned with their original functions, thereby eliciting unforeseen cellular responses (1). These distinctive structures are designated as alarmins – small, endogenous immune-activating peptides or proteins that are released in response to cellular injury or death. Primarily, alarmins serve non-immunological roles within the cellular environment, including the protection of proteins from acute denaturation and aggregation, as well as binding to chromatin, thereby functioning as transcriptional regulators (1, 2). According to comprehensive studies, homeostatic intracellular proteins can also perform non-homeostatic roles in the extracellular environment, signaling danger and promoting inflammation (3); thus, they are designated as danger-associated molecular patterns (DAMPs) (4). During trauma, DAMPs may serve as adjuvants by engaging with pattern recognition receptors (PRRs) to convey “danger” signals to the host and initiate a local inflammatory response, which facilitates tissue regeneration (4, 5). An inflammatory response following sterile tissue injury and the subsequent repair mechanisms may hold comparable significance for the evolutionary fitness of a multicellular organism as inflammation induced by infectious microorganisms (6). Although a sterile immune response is essential for preserving physiological homeostasis, chronic and extended inflammation may interfere with cellular processes, cause cell damage, and ultimately result in cell death (7).

High-mobility group box 1 (HMGB1) is an evolutionarily ancient and ubiquitous nuclear protein present in the majority of cell types (8, 9), recognized as the first member of multifunctional alarmins (1, 10). The functions of HMGB1 depend on the location of the molecule, its binding partners, and its redox state (9). Within the nucleus, HMGB1 is responsible for organizing DNA and nucleosomes while also regulating gene transcription. Research indicates that substantial quantities of HMGB1 are passively released from the nucleus into the extracellular space immediately following an injury (11). Nuclear HMGB1 is capable of translocating to the cytoplasm, where it plays a role in the activation of the inflammasome, pyroptosis, and the regulation of the autophagy/apoptosis balance (12). HMGB1 is also implicated in the recently characterized form of cell death known as cuproptosis, which is induced by copper-dependent mitochondrial stress within cells. In cuproptotic cells lacking HMGB1, the ability to induce receptor for advanced glycation end products (RAGE)-dependent proinflammatory cytokine production is significantly impaired, suggesting that HMGB1 functions as a key immune mediator in sterile inflammation (10, 13). The ability of HMGB1 to be actively secreted, as well as passively released, provides a mechanism for emitting a general alarm signal in the presence of significant cellular stress, without necessarily implying cellular damage (9, 12). Several studies have identified HMGB1 as a component of neutrophil extracellular traps (NETs) (14) and its interactions with a variety of molecules, including lipopolysaccharides (LPS), interleukin-1β (IL-1β), single-stranded DNA, peptidoglycan (PGN), and nucleosomes. The subsequent complexes formed enhance the inflammatory response by binding to the receptors of their interacting partners in each respective complex (15, 16).

Mast cells (MCs), a type of immune cell found in all classes of vertebrates, play a pivotal role in maintaining tissue function and integrity (17). These cells are widely dispersed throughout nearly all tissues and are characteristically located in close proximity to epithelial cells, fibroblasts, blood vessels, lymphatic vessels, and nerves (18). The specific distribution, attributes, and phenotypic plasticity of resident MCs substantially contribute to the adaptability of various biological processes and the preservation of homeostasis (19, 20). They are associated with numerous physiological and inflammatory processes (21), encompassing organ development, wound healing, angiogenesis, lymphangiogenesis (22), cardiac function (23), and host defense mechanisms (24). Additionally, MCs fulfill an essential role in every phase of tissue repair, beginning with the initial inflammatory response and extending through the remodeling of the extracellular matrix (ECM) (25). It is imperative to recognize that, following an injury, MCs assume a pivotal role in regulating primary hemostasis to seal the damaged surface effectively (26, 27). These cells function as the primary effectors of inflammation; they appear to have evolved as cellular sensors capable of discerning their environment to initiate an appropriate physiological response, which may either promote inflammation for repair or, conversely, limit the inflammatory process to prevent further damage (28, 29).

Given the substantial role of MCs in both physiological and pathological processes, it is essential to identify and characterize the endogenous factors that exert influence over their biology and functionality. The available information regarding the impact of HMGB1 on mature MCs and its significance in modulating MC activity within tissues remains inadequate. In light of these findings, the present study investigates whether HMGB1 affects the expression levels of specific PRRsin mature MCs. These receptors include Dectin-1 and Dectin-2, which are categorized as C-type lectin receptors (CLRs), TLR2 from the class of Toll-like receptors (TLRs), NOD1 from nucleotide-binding oligomerization domain-like receptors (NLRs), and RIG-I from retinoic acid-inducible gene-I-like receptors (RLRs). Furthermore, this study aims to ascertain whether HMGB1 modulates the inflammatory response of these cells, which encompasses the production of cytokines, chemokines, reactive oxygen species (ROS), histamine, and cysteinyl leukotrienes (cysLTs), along with their migration patterns. The study intends to examine the role of RAGE in HMGB1-mediated MC activation, with a specific emphasis on the participation of signaling molecules such as phosphoinositide 3-kinase (PI3K), extracellular signal-regulated kinase (ERK) 1/2, mitogen‐activated protein kinase p38, nuclear factor kappa B (NF-κB), and Janus‐activated kinase (JAK) 2.

## 2 Materials and methods

### 2.1 Isolation of MCs

The study was conducted on female albino Wistar rats (Crl: WI; Charles River Laboratories, Wilmington, MA, USA) with an average weight of approximately 250 grams and an age range of 3 to 4 months. The animals were procured from the animal quarters of the Faculty of Biology and Environmental Protection at the University of Lodz. The experimental protocols received approval from the Local Ethics Committee for Experiments on Animals in Lodz (no 15/2021). Every effort was undertaken to minimize animal suffering. Prior to decapitation, all animals were anesthetized with isoflurane (BAXTER, Deerfield, IL, USA). Peritoneal cell suspensions were obtained using lavage with 50 mL of 1% Hank’s Balanced Salt Solution (HBSS; GIBCO, Gaithersburg, MD, USA) supplemented with 0.015% sodium bicarbonate (GIBCO). Following an abdominal massage of approximately 90 seconds, the cell suspension was extracted from the peritoneal cavity. The peritoneal cell suspension underwent two washes (150 x g, 5 minutes, 20°C) in complete Dulbecco’s Modified Eagle Medium (cDMEM), which consisted of DMEM (Biowest, Kansas City, MO, USA) supplemented with 10% fetal calf serum (FCS; GIBCO), 10 μg/mL gentamicin (GIBCO), and 2 mM glutamine (GIBCO). Isotonic 72.5% Percoll (Sigma-Aldrich, St. Louis, MO, USA) density gradient centrifugation (190 x g, 15 minutes, 20°C) was applied for the purification of MCs. Subsequently, the isolated MCs were centrifuged twice in cDMEM (150 x g, 5 minutes, 20°C). The isolation process for MCs lasted approximately 45 to 50 minutes. After washing, MCs were counted and resuspended in an appropriate volume of cDMEM (for quantitative RT-PCR, flow cytometry, and confocal microscopy analysis, as well as migration assays) or a medium specifically designed for rat MCs, which contained 137 mM NaCl (Sigma-Aldrich), 2.7 mM KCl (Sigma-Aldrich), 1 mM MgCl2 (Sigma-Aldrich), 1 mM CaCl2 (Sigma-Aldrich), 10 mM HEPES (Sigma-Aldrich), 5.6 mM glucose (Sigma-Aldrich), and 1 mg/mL bovine serum albumin (BSA; Sigma-Aldrich) (for histamine release assays, ELISA assays, and ROS generation), to achieve a MC concentration of 1.5 × 10^6^ cells/mL. A suitable number of animals was employed to attain the appropriate MC density and the required number of samples in a specific type of experiment. The MCs were prepared with a purity exceeding 98%, as measured by metachromatic staining using toluidine blue (Sigma-Aldrich). The viability of MCs was greater than 98%, determined through the trypan blue (Sigma-Aldrich) exclusion assay. The results obtained from the treated samples were compared to the control samples within the confines of the respective experiment.

### 2.2 Quantitative RT-PCR

Quantitative reverse transcription polymerase chain reaction (qRT-PCR) was employed to assess constitutive and HMGB1-induced mRNA levels of receptors and cytokines/chemokines in MCs. Purified MCs, suspended in cDMEM, were stimulated with HMGB1 (Novus Biologicals, Centennial, CO, USA) at a final concentration of 1 μg/mL for 2 hours at 37°C in a humidified atmosphere containing 5% CO_2_. For the control group, MCs were maintained under identical conditions without HMGB1. Total RNA was extracted from the cells utilizing the RNeasy^®^ Mini Kit (Qiagen, Valencia, CA, USA). Subsequently, complementary DNA (cDNA) was synthesized according to the manufacturer’s protocol for the iScript cDNA Synthesis Kit (Bio-Rad Laboratories, Hercules, CA, USA). qRT-PCR was conducted utilizing the CFX96 Touch™ Real-Time PCR Detection System (Bio-Rad Laboratories) in conjunction with the iTaq™ Universal SYBR^®^ Green Supermix (Bio-Rad Laboratories). The volume of the PCR reaction comprised 5 µL of iTaq™ Universal SYBR^®^ Green Supermix, 1 μL of cDNA, 2 μL of primers (500 nM), and 2 μL of PCR-grade water provided within the kit. The cycling conditions were established as follows: an initial denaturation at 95°C for 3 minutes, followed by 40 cycles consisting of denaturation at 95°C for 10 seconds, annealing at 60°C for 10 seconds, and extension at 72°C for 20 seconds. The fold changes in the tested samples were calculated using the Bio-Rad CFX Maestro Software, employing the ΔΔCt method. The expression levels of the receptor and cytokine/chemokine mRNAs were normalized based on the transcript level of the housekeeping gene, rat *Actb*. Unstimulated specimens were utilized as calibrator samples. The primer sequences are delineated in **Table 1**.

**Table 1:** Sequences of primers used in the study.

Gene name	Primer sequence (5’-3’)
*ACTB*	Forward: TCTGTGTGGATTGGTGGCTCTA Reverse: CTGCTTGCTGATCCACATCTG
*CCL2*	Forward: ATGCAGTTAATGCCCCACTC Reverse: TTCCTTATTGGGGTCAGCAC
*CCL3*	Forward: CATGGCGCTCTGGAACGAA Reverse: TGCCGTCCATAGGAGAAGCA
*CCL4*	Forward: TATGAGACCAGCAGCCTTTGC Reverse: GCACAGATTTGCCTGCCTTT
*DECTIN1*	Forward: TGGACGAAGATG GATATAC Reverse: CAAGCACAGGATTCCTA
*DECTIN2*	Forward: GCTAGCTGCGTGATTTCCA Reverse: TGAAACACACCGCTCTTCTG
*GMCSF*	Forward: AGACCCGCCTGAAGCTATACAA Reverse: CTGGTAGTGGCTGGCTATCATG
*IFNA*	Forward: CTGCTGTCTAGGATGTGACCTGC Reverse: TTGAGCCTTCTGGATCTGCTG
*IFNB*	Forward: CGTTCCTGCTGTGCTTCTC Reverse: TGTAACTCTTCTCCATCTGTGAC
*IFNG*	Forward: ACGCCGCGTCTTGGTTT Reverse: AGGCTTTCAATGAGTGTGCTT
*IL1B*	Forward: CACCTCTCAAGCAGAGCACAG Reverse: GGGTTCCATGGTGAAGTCAAC
*IL4*	Forward: ATGCACCGAGATGTTTGTACC Reverse: TTTCAGTGTTCTGAGCGTGGA
*IL6*	Forward: TCCTACCCCAACTTCCAATGCTC Reverse: TTGGATGGTCTTGGTCCTTAGCC
*IL10*	Forward: CACTGCTATGTTGCCTGCTC Reverse: TTCATGGCCTTGTAGACACC
*IL18*	Forward: AAACCCGCCTGTGTTCGA Reverse: ATCAGTCTGGTCTGGGATTCGT
*IL33*	Forward: TCGCACCTGTGACTGAAATC Reverse: ACACAGCATGCCACAAACAT
*NOD1*	Forward: GTCCTCAACGAGCATGGCGAGACT Reverse: TGCAGCTCATCCAGGCCGTCAA
*RIGI*	Forward: AAAGCCAGAGACCAAGACCA Reverse: TATCTCCGCTGGCTCTGAAT
*TGFB*	Forward: CGTGGAAATCAATGGGATCAG Reverse: GGAAGGGTCGGTTCATGTCA
*TLR2*	Forward: GTACGCAGTGAGTGGTGCAAGT Reverse: GGCCGCGTCATTGTTCTC
*TNF*	Forward: AAATGGGCTCCCTCTCATCAGTTC Reverse: TCTGCTTGGTGGTTTGCTACGAC

### 2.3 Cell preparation for flow cytometric and confocal microscopy analysis

The expression levels of Dectin-1, Dectin-2, TLR2, NOD1, and RIG-I induced by HMGB1, as well as their constitutive expression, were evaluated through flow cytometry and confocal microscopy. The constitutive expression of Dectin-1, Dectin-2, TLR2, NOD1, and RIG-I was analyzed in unstimulated MCs. Induced receptor expression was assessed in MCs that were incubated with HMGB1 at a final concentration of 10 ng/mL for durations of 1 or 3 hours at 37°C in a humidified atmosphere containing 5% CO_2_. To ascertain the intracellular localization of the receptors, the MCs were fixed using CellFIX™ (BD Bioscience, San Jose, USA) solution for 15 minutes at 4°C and subsequently washed twice with 1 × PBS (Cayman Chemical, Ann Arbor, USA). Following this, the MCs were permeabilized with 0.1% saponin (Sigma-Aldrich) for 30 minutes at room temperature. The MCs were then resuspended in 1 × PBS and stained for 1 hour with goat anti-Dectin-1, goat anti-Dectin-2 (Invivogen, San Diego, CA, USA), rabbit anti-TLR2, goat anti-NOD1, or goat anti-RIG-I antibodies (Santa Cruz Biotechnology, Inc., Dallas, USA) at a dilution of 1:100. For the control, MCs were stained with goat or rabbit IgG isotype control (R&D Systems, Minneapolis, USA) exhibiting irrelevant specificity. The primary antibody was omitted from the sample to confirm the non-specific binding of the secondary antibody. Subsequently, the cells were washed with 1 × PBS and incubated with Alexa Fluor 488^®^ rabbit anti-goat IgG or Alexa Fluor 488^®^ goat anti-rabbit IgG (Jackson ImmunoResearch Laboratories, Inc., West Grove, USA) at a dilution of 1:100 in 1 × PBS for 1 hour in the dark. To establish the surface localization of the receptors, the MCs were fixed with CellFIX™ (BD Bioscience, San Jose, USA) solution for 15 minutes at 4°C and washed twice with 1 × PBS (Cayman Chemical, Ann Arbor, USA). Following this procedure, the cells were washed twice and finally resuspended in 1 × PBS prior to receptor assessment. After each incubation period, the viability of the MCs was evaluated using the trypan blue exclusion test.

### 2.4 Flow cytometry

A total of ten thousand events from each sample were analyzed utilizing a BD FACSCalibur™ flow cytometer equipped with BD CellQuest™ software (BD Biosciences). The expression of HMGB1-dependent MC Dectin-1, Dectin-2, TLR2, NOD1, and RIG-I was presented as a percentage of the mean fluorescence intensity (MFI) of Dectin-1/Dectin-2/TLR2/NOD1/RIG-I, measured in unstimulated MCs, which is referred to as 100%.

### 2.5 Confocal microscopy

The samples were affixed to microscope slides, and images were acquired using a Leica TCS SP8 microscope (Wetzlar, Germany) equipped with the HC PL APO CS2 63x/1.4 oil objective at the Laboratory of Microscopic Imaging and Specialized Biological Techniques at the University of Lodz. A 488 nm laser was employed to excite the fluorescence, and the emission was collected by a hybrid detector within the range of 505–550 nm. To facilitate visualization of the cells, the PMT transmission channel was utilized. LAS X 2.0.2.15022 software (Leica Microsystems, Wetzlar, Germany) was used for data analysis. All settings were maintained consistently throughout the experiments. All signals obtained from confocal microscopy were verified through profile view image analysis, along with diagrams presenting intensity values positioned beneath each microphotograph. The mean fluorescence intensity (expressed in arbitrary units, AU) was calculated for each sample. The calculations were conducted for a minimum of 40 different points, randomly selected within compartments exhibiting receptor expression.

### 2.6 ELISA

In order to measure the generation of cytokines and chemokines, purified MCs suspended in a medium designed for rat MCs were incubated with HMGB1 at final concentrations of 0.1, 1, and 10 μg/mL, or with buffer alone to assess spontaneous cytokine and chemokine generation. This incubation occurred in a humidified environment containing 5% CO_2_ for a duration of 3 hours at a temperature of 37°C. Subsequently, the supernatants were collected through centrifugation. The concentrations of TNF, TGF-β, IL-1β, CCL3, CCL4, and cysLTs in the supernatants were determined using ELISA kits – specifically, TNF and IL-1β from Wuhan Eiaab Science Inc., Wuhan, China; TGF-β, CCL3, and CCL4 from Biorbyt Ltd., Cambridge, UK; and cysLTs from Cayman Chemical, Michigan, USA—following the manufacturer’s instructions. The sensitivity of these assays was determined to be < 7 pg/mL, < 6 pg/mL, < 14 pg/mL, < 1 pg/mL, < 15 pg/mL, and < 20 pg/mL, respectively.

### 2.7 Histamine-release assay

Purified MCs suspended in the medium specifically designed for rat MCs were incubated with HMGB1 at final concentrations of 0.1, 1, and 10 μg/mL, and compound 48/80 (Sigma-Aldrich), a well-established potent MC degranulation factor (30), at a final concentration of 5 µg/mL (positive control) or buffer alone (spontaneous histamine release) in a water bath for 30 minutes at 37°C with continuous stirring. Following the incubation period, the reaction was halted by adding 1.9 mL of cold medium. Subsequently, the cell suspension was subjected to centrifugation, and the supernatants were transferred into separate tubes. A total of 2 mL of distilled water was incorporated into each tube containing the cell pellets. The histamine content was quantified in both the cell pellets (residual histamine) and supernatants (released histamine) utilizing the spectrofluorometric method, as previously detailed (31). The histamine release was expressed as a percentage of the total cellular content of the amine.

### 2.8 Migration assay

The MC migratory response to HMGB1 was investigated utilizing a Boyden microchamber assay (Neuro Probe, Gaithersburg, USA) within a 48-well chemotaxis chamber (Neuro Probe). Thirty microliters of HMGB1 at final concentrations of 0.1, 1, and 10 μg/mL, or buffer alone (control for spontaneous migration), were placed into the lower compartments of the microchamber. These lower compartments were then covered with a polycarbonate membrane featuring an 8-μm pore size, and 50 μL of the cell suspensions were introduced into the upper compartments. Subsequently, the chemotaxis chamber was incubated for 3 hours in a humidified atmosphere containing 5% CO_2_ at 37°C. After the incubation period, MCs that adhered to the upper surface of the membrane were removed by gently scraping with a rubber blade. Migrating cells that adhered to the lower surface of the membrane were fixed in 99.8% ethanol (Avantor Performance Materials, Poland), stained for 10 minutes with hematoxylin (Sigma-Aldrich), cleared in distilled water, and then mounted on a microscope slide. MC migration was quantified by counting the number of cells that traversed the membrane and adhered to the bottom surface of the filter. Ten high-power fields (HPF) were assessed in each assay (x 250). The spontaneous migration served as a control and was designated as 100%. The results were presented as a percentage of the control migration.

### 2.9 Measurement of intracellular ROS production

To determine the generation of reactive oxygen species (ROS), MCs suspended in a medium formulated for rat MCs were incubated with HMGB1 at final concentrations of 0.1, 1, and 10 μg/mL, or with the medium alone, for a duration of 1 hour within a humidified atmosphere containing 5% CO2 at 37°C. Subsequently, CellROX™ Green Reagent (Invitrogen, Carlsbad, CA, USA) was introduced at a final concentration of 5 µM and incubated with the cells at 37°C for an additional 30 minutes. The cells were then subjected to three washing cycles with 1 × PBS and subsequently analyzed using the FLUOstar Omega Microplate Reader (BMG Labtech, Ortenberg, Germany). The fluorescence intensity was assessed at excitation and emission wavelengths of 485/520 nm. ROS generation was quantified and expressed as mean signal intensity (MSI).

### 2.10 RAGE receptor involvement

To ascertain the role of the HMGB1 receptor, the RAGE antagonist FPS-ZM1 (R&D Systems) was employed. Purified MCs underwent pretreatment with the antagonist for 15 minutes at 37°C in a water bath with continuous stirring prior to the execution of the principal procedures, which included ELISA for protein measurements, the histamine-release assay, the kit for intracellular ROS production measurement, and the migration assay. FPS-ZM1 was utilized at a concentration of 5 μM. The concentration of the antagonist was selected based on preliminary experiments conducted in accordance with the manufacturer’s guidelines.

### 2.11 MC treatment with signaling pathway inhibitors

For the analysis of cellular signaling pathways, purified MCs were pre-treated with various signaling molecule inhibitors or medium alone for 1 hour at 37°C prior to the commencement of the primary procedure. ERK1/2 inhibitor PD98059 (Sigma-Aldrich) was administered at a concentration of 50 μM, p38 inhibitor SB203580 (Sigma-Aldrich) at 10 μM, PI3K inhibitor LY294002 (Sigma-Aldrich) at 50 μM, and JAK2 inhibitor AG490 (Merck Millipore, Billerica, MA, USA) was used at 10 μM. MCs were also pre-incubated with 3 μM of NF-κB inhibitor MG-132 (InvivoGen) for 15 minutes at 37°C. The concentrations of all applied inhibitors were determined during preliminary experiments, in accordance with the manufacturer’s instructions. None of the inhibitors compromised MC viability, as confirmed by the trypan blue exclusion assay. Following incubation, the MCs were washed twice in 1 × PBS, re-suspended in appropriate medium, and utilized in the histamine-release assay, migration assay, and measurement of intracellular ROS production, as previously described.

### 2.12 Statistical analysis

The statistical analysis of the experimental data was conducted utilizing Statistica 13 software (Statsoft Inc., USA). The data are represented as mean ± standard deviation (SD). The normality of distribution was assessed using the Shapiro-Wilk test. The Student’s *t*-test and one-way ANOVA followed by *post hoc* Tukey’s test were employed to assess significant differences, with a *P*-value of < 0.05 regarded as statistically significant. The adjusted *P*-values are presented in **Supplementary Data Tables S1**–**S3**.

## 3 Results

### 3.1 HMGB1 impacts on Dectin-1, Dectin-2, TLR2, NOD1, and RIG-I expression in MCs

**Figure 1:**
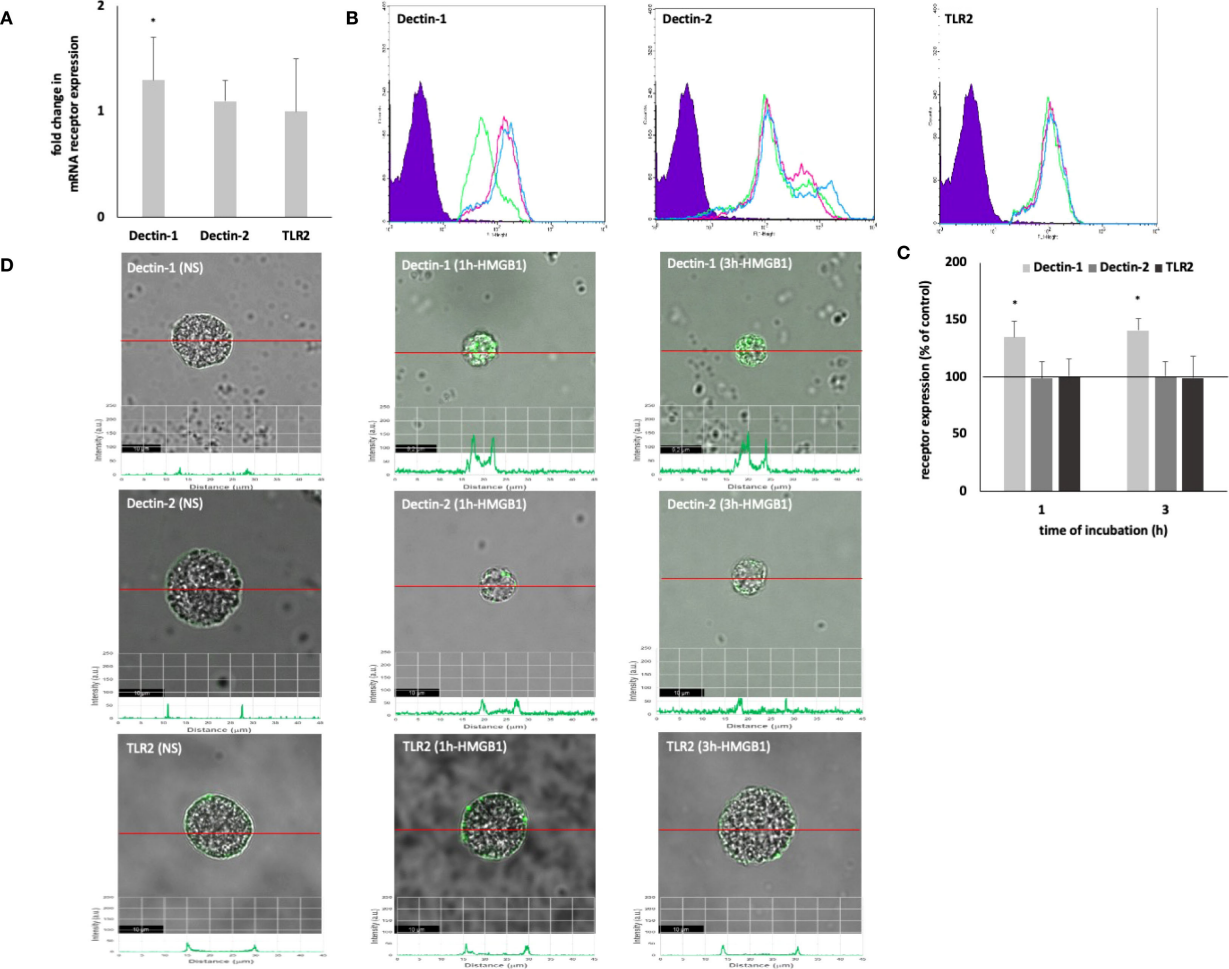
Constitutive and HMGB1-induced Dectin-1/Dectin-2/TLR2 mRNA and protein expression in MCs. Cells were incubated with medium alone (non-stimulated MCs; NS) or HMGB1 at 10 ng/mL, and receptor expression was assessed by **(A)** qRT-PCR, **(B, C)** flow cytometry, and **(D)** confocal microscopy. **(B)** Representative flow cytometry histograms showing Dectin-1, Dectin-2, and TLR2 expression after HMGB1 stimulation in non-permeabilized cells. Shaded tracings – isotype control, open tracings – receptor expression in unstimulated MCs (green) and after HMGB1 stimulation for 1 h (violet) and 3 h (blue). **(C)** Constitutive receptor expression served as a control and was referred to as 100%. The results are presented as a percentage of constitutive receptor expression. Results are the mean of fluorescent intensity ± SD of three experiments performed in duplicate. Differences were considered significant at *P* < 0.05 and are labelled with an asterisk (*) on each graph (Student’s *t*-test), P* < 0.05. **(D)** Representative images showing Dectin-1/Dectin-2/TLR2 surface localization in non-permeabilized non-stimulated (NS) and HMGB1-stimulated MCs analyzed by confocal microscopy.

Initially, the capacity of HMGB1 to modulate the expression of PRRs on MCs was investigated. The qRT-PCR technique was employed to ascertain the mRNA expression levels of Dectin-1, Dectin-2, and TLR2 in unstimulated MCs compared to those stimulated with HMGB1. As illustrated in **Figure 1A**, HMGB1 led to a 1.3-fold upregulation of Dectin-1 mRNA expression (*P* < 0.05), while it did not affect the mRNA levels of Dectin-2 and TLR2. Subsequently, we explored whether HMGB1 influences the baseline expression levels of the selected surface PRRs utilizing flow cytometry and confocal microscopy. Receptor expression was assessed in unstimulated MCs and those exposed to HMGB1 (10 ng/mL) for durations of 1 hour or 3 hours. We observed that the baseline level of Dectin-1 expression was significantly (*P* < 0.05) upregulated following incubation with HMGB1 for both 1 hour and 3 hours, reaching values of 138.84 ± 13.42% and 140.56 ± 10.81% relative to control Dectin-1 expression, respectively (**Figures 1B, C**). Confocal and fluorescence intensity imaging indicated that cell surface signals for Dectin-1 peaked at 131.41 ± 5.81 arbitrary units (A.U.) (*P* < 0.05) after 1 hour and 133.15 ± 11.23 A.U. (*P* < 0.05) after 3 hours of incubation (**Figure 1D**). We established statistically significant differences determined by one-way ANOVA (*P* < 0.0001), too. Additionally, flow cytometry and confocal microscopy validated the presence of Dectin-2 and TLR2 on the surface of non-stimulated (NS) MCs, revealing no significant alterations in expression levels following stimulation with HMGB1.

**Figure 2:**
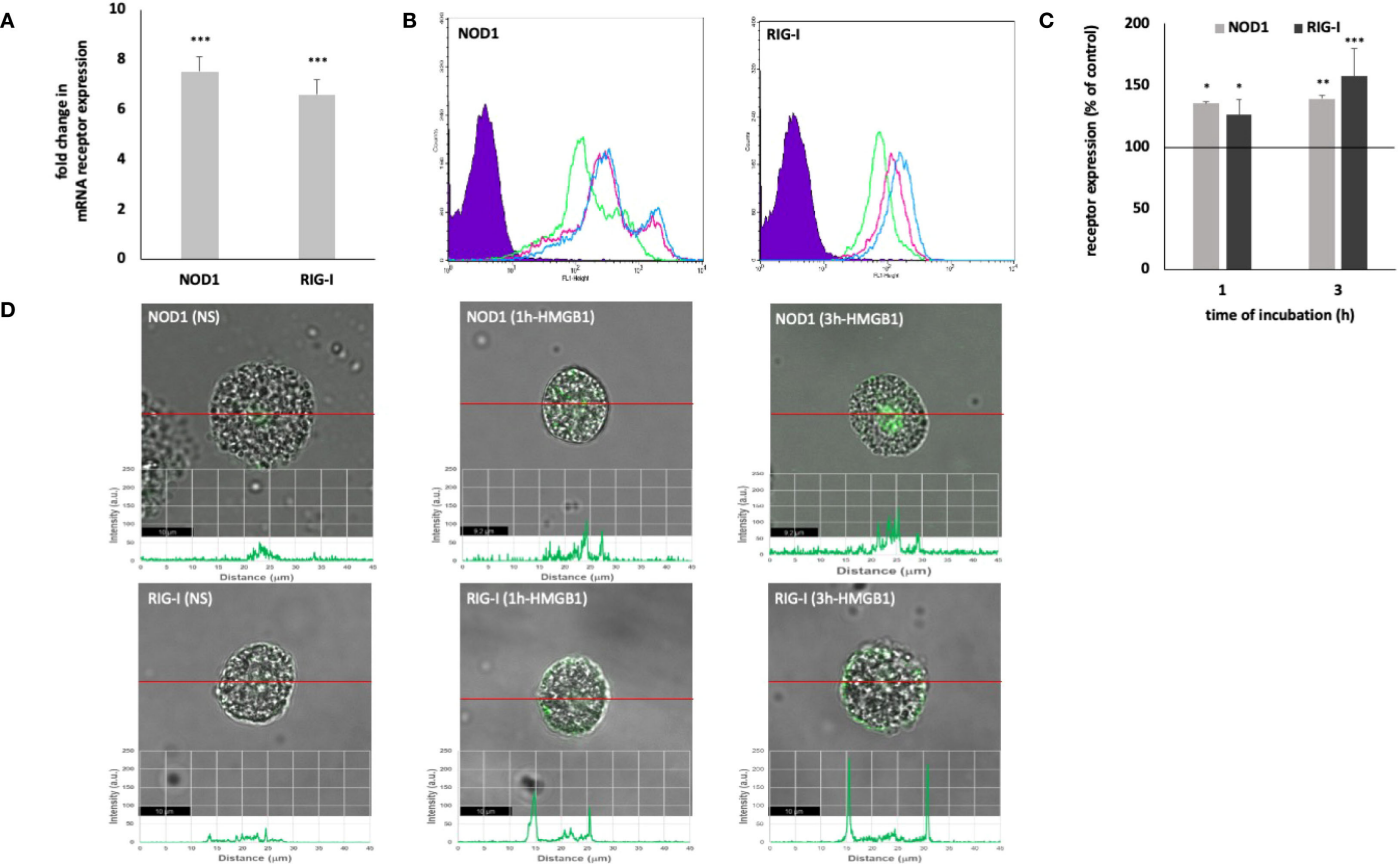
Constitutive and HMGB1-induced NOD1 and RIG-I mRNA and protein expression in MCs. Cells were incubated with medium alone (non-stimulated MCs; NS) or HMGB1 at 10 ng/mL, and receptor expression was assessed by **(A)** qRT-PCR, **(B, C)** flow cytometry, and **(D)** confocal microscopy. **(B)** Representative flow cytometry histograms showing NOD1 and RIG-I expression after HMGB1 stimulation in permeabilized cells. Shaded tracings – isotype control, open tracings – receptor expression in unstimulated cells (green) and after HMGB1 stimulation for 1 h (violet) and 3 h (blue). **(C)** Constitutive receptor expression served as a control and was referred to as 100%. The results are presented as a percentage of constitutive receptor expression. Results are the mean of fluorescent intensity ± SD of three experiments performed in duplicate. Differences were considered significant at *P* < 0.05 and are labeled with an asterisk (*) on each graph (Student’s *t*-test), P* < 0.05, P** < 0.01, P*** < 0.001. **(D)** Representative images showing NOD1/RIG-I cellular localization in permeabilized non-stimulated (NS) and HMGB1-stimulated MCs, analyzed by confocal microscopy.

The expression of intracellular PRRs by MCs – NOD1 and RIG-I is shown in **Figure 2**. HMGB1 strongly affected both NOD1 (*P* < 0.001) and RIG-I (*P* < 0.001) mRNA expression (**Figure 2A**). HMGB1 induced enhancement of the intracellular NOD1 protein level, and the intensity of the signals reaching after 1 h 134.78 ± 1.30% (*P* < 0.05) and after 3 h 138.98 ± 2.42% (*P* < 0.01) of control receptor expression (**Figures 2B, C**). Using immunocytochemical staining, we visualized the presence of NOD1, mainly in the nuclear region (**Figure 2D**). Image analysis revealed that the receptor expression was upregulated upon incubation with this molecule (NS: 33.86 ± 6.56; HMGB1: 1h - 70.72 ± 15.17 A.U., 3h - 94.99 ± 15.57 A.U.) which was confirmed by a one-way ANOVA (*P* = 0.0026). The signals were strongly associated with the cytoplasm located near the cell nucleus.

The one-hour incubation of MCs with HMGB1 resulted in a statistically significant increase in the intracellular RIG-I level, with a *P*-value of less than 0.05, when compared to the control group of unstimulated MCs (**Figures 2B, C**). Extended incubation further enhanced the expression of the receptor, achieving 156.95 ± 26.17% of the baseline level, with a p-value of less than 0.001. Stimulation of MCs with HMGB1 led to an increase in RIG-I expression, as evidenced by intensity diagrams accompanying each microphotograph (NS: 23.17 ± 4.88; HMGB1: 1 h - 117.67 ± 11.30 A.U., 3 h - 200.34 ± 17.40 A.U.) (refer to **Figure 2D**). We also found statistically significant differences, as determined by one-way (*P* < 0.0001). Significant enrichment in the signals was noted in the intracellular regions and beneath the cell surface.

### 3.2 HMGB1 effects on MC pro-inflammatory response

**Figure 3:**
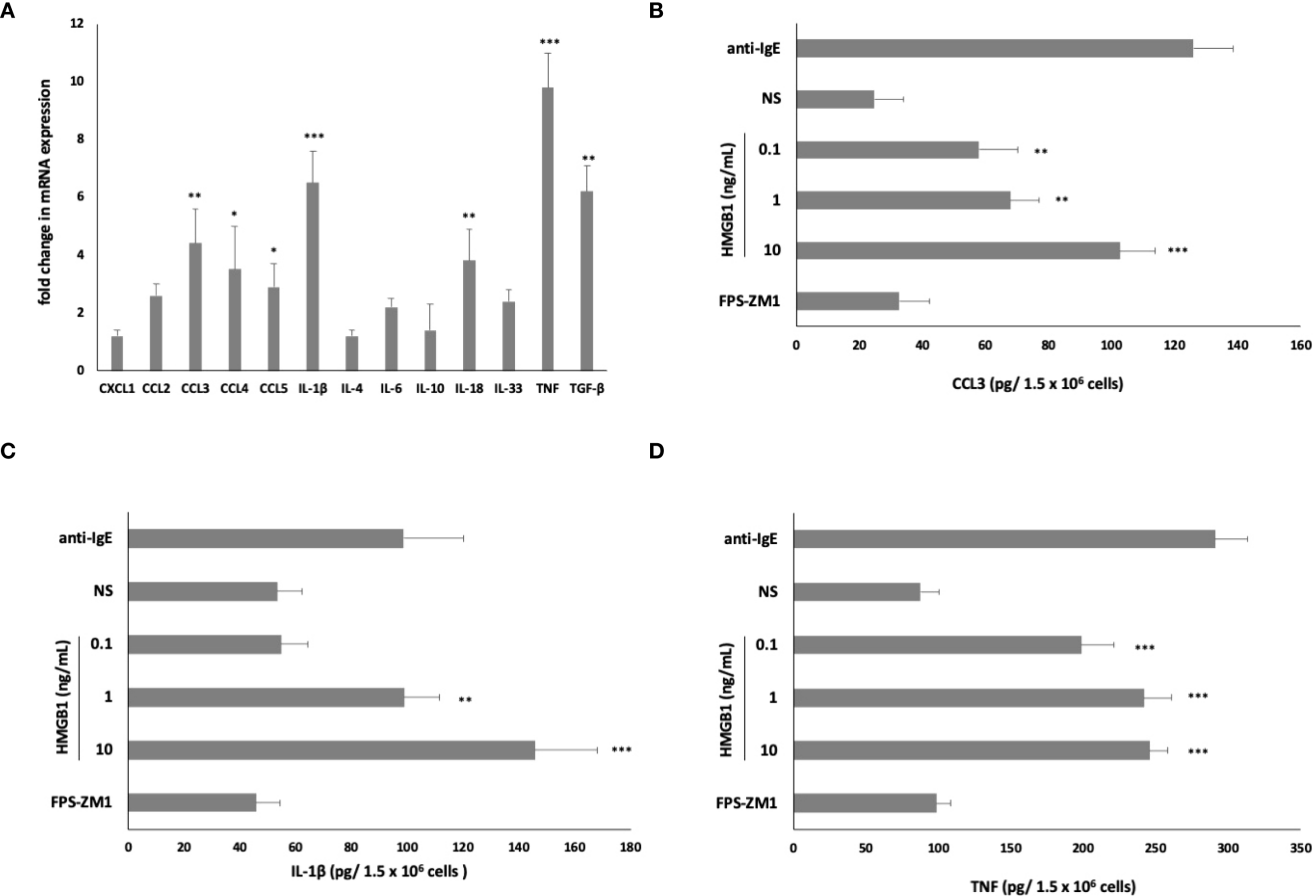
Effect of HMGB1 on **(A)** chemokine/cytokine mRNA expression, **(B)** CCL3, **(C)** IL-1β, and **(D)** TNF protein synthesis in MCs. For mRNA measurement, MCs were incubated with HMGB1 at 10 ng/mL or medium alone. For protein measurement, MCs were incubated with HMGB1 at 0.1–10 ng/mL, anti-IgE at 5 μg/mL (positive control) or medium alone (non-stimulated MCs; NS). RAGE antagonist FPS-ZM1 was used at 5 μM before the MCs incubation with 10 ng/mL HMGB1 and protein evaluation. Results are the mean ± SD of four independent experiments. Differences were considered significant at *P* < 0.05 and are labelled with an asterisk (*) on each graph (Student’s *t*-test)), P* < 0.05, P** < 0.01, P*** < 0.001.

**Figure 4:**
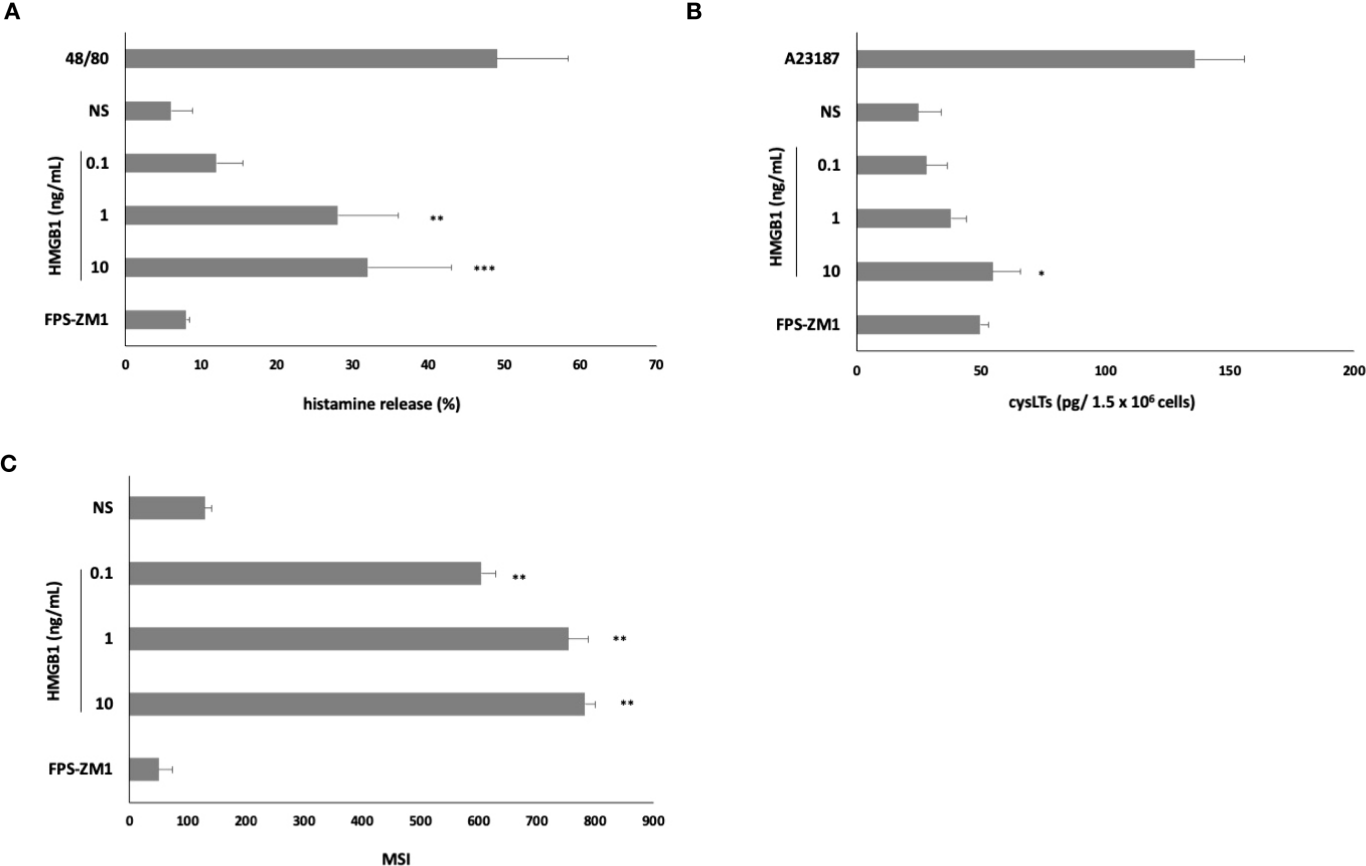
Effect of HMGB1 on **(A)** histamine release, **(B)** cysLT synthesis and release, and **(C)** ROS production by MCs. To the histamine assessment, cells were incubated with HMGB1 at 0.1–10 ng/mL, compound 48/80 at 5 μg/mL (positive control), or medium alone (non-stimulated MCs; NS) for 30 min. For cysLTs assessment, MCs were incubated with HMGB1 at 0.1–10 ng/mL, calcium ionophore A23187 at 5 μg/mL (positive control), or medium alone (non-stimulated MCs; NS). To evaluate ROS generation, MCs were incubated with HMGB1 at 0.1–10 ng/mL or medium alone (NS). RAGE antagonist FPS-ZM1 was used at 5 μM before the MCs incubation with 10 ng/mL HMGB1. Results are the mean ± SD of three independent experiments, and each experiment was performed in duplicate. Differences were considered significant at *P* < 0.05 and are labeled with an asterisk (*) on each graph (Student’s *t*-test), P* < 0.05, P** < 0.01, P*** < 0.001.

Subsequently, to determine whether HMGB1 could elicit an inflammatory and immunoregulatory response from MCs, we assessed this factor’s capacity to influence the expression of chemokine/cytokine mRNA. qRT- PCR was conducted, and the fold change in cytokine/chemokine mRNA expression in HMGB1-stimulated (10 ng/mL) MCs compared to non-stimulated cells was evaluated. As illustrated in **Figure 3A**, among the chemokines/cytokines analyzed in HMGB1-stimulated MCs, the highest levels of mRNA expression were noted for TNF (9.8-fold increase), IL-1β (6.5-fold increase) (*P* < 0. 001), TGF-β (6.2-fold change), CCL3 (4.4-fold change), IL-18 (3.8- fold change) (*P* < 0. 01), CCL4 (3.5-fold change), and CCL5 (2.9-fold change) (*P* < 0. 05). In the subsequent phase, we investigated one chemokine and two cytokines exhibiting the greatest mRNA level increases for protein synthesis. For this purpose, MCs were stimulated with HMGB1 at concentrations ranging from 0.1 to 10 ng/mL for 3 hours, utilizing medium alone as a negative control and anti-IgE as the positive control. Additionally, our objective was to clarify the role of RAGE in the activation of MCs by HMGB1. The results of these experiments are presented in **Figures 3B–D**. The most pronounced secretion of the CCL3 chemokine was observed at 10 ng/mL of HMGB1, which increased to 102.56 ± 11.23 pg/1.5 × 10^6^ cells (*P* < 0.001; ANOVA *P* = 0.0057). Furthermore, we observed that HMGB1 also stimulated MCs to produce CCL3 at lower concentrations of 0.1 and 1 ng/mL (*P* < 0. 01) (**Figure 3B**). The statistical analysis indicated that HMGB1 at 10 ng/mL was the most potent inducer of IL-1β production (*P* < 0.05; ANOVA *P* = 0.0185) (**Figure 3C**). Conversely, in the case of MCs stimulated with HMGB1 across all concentrations, we recorded a significantly higher release of TNF (*P* < 0.001; ANOVA *P* = 0.0006) compared to non-stimulated cells (**Figure 3D**). To ascertain whether RAGE is implicated in the HMGB1-mediated response in MCs, the receptor antagonist FPS-ZM1 was employed. As demonstrated in **Figures 3B–D**, the pretreatment of MCs with FPS-ZM1 inhibited HMGB1-induced CCL3/IL-1β/TNF generation. We also established that HMGB1 at 1 (*P* < 0.01) and 10 (*P* < 0.001) ng/mL activated MCs to degranulation, assessed by histamine secretion (**Figure 4A**). There were statistically significant differences between means as determined by one-way ANOVA in this case (*P* < 0.0001). As shown in **Figure 4B**, HMGB1 did not stimulate cysLT production and release by MCs at 0.1 and 1 ng/mL but induced this mediator’s generation at 10 ng/mL (*P* < 0.05). Additionally, spectrofluorimetry was used to examine ROS generation by MCs in response to stimulation with HMGB1. As demonstrated in **Figure 4C**, MCs produced significant amounts of ROS after stimulation with HMGB1. Of the various molecule concentrations, the most significant ROS generation was observed at 10 ng/mL HMGB1, rising to 782.3 ± 18.45 MSI. We observed statistically significant differences, as determined by one-way ANOVA (*P* < 0.0001). Additionally, preincubation of MCs with FPS-ZM1 resulted in inhibition of HMGB1-induced histamine and ROS generation.

### 3.3 HMGB1 impacts on MC migration

**Figure 5:**
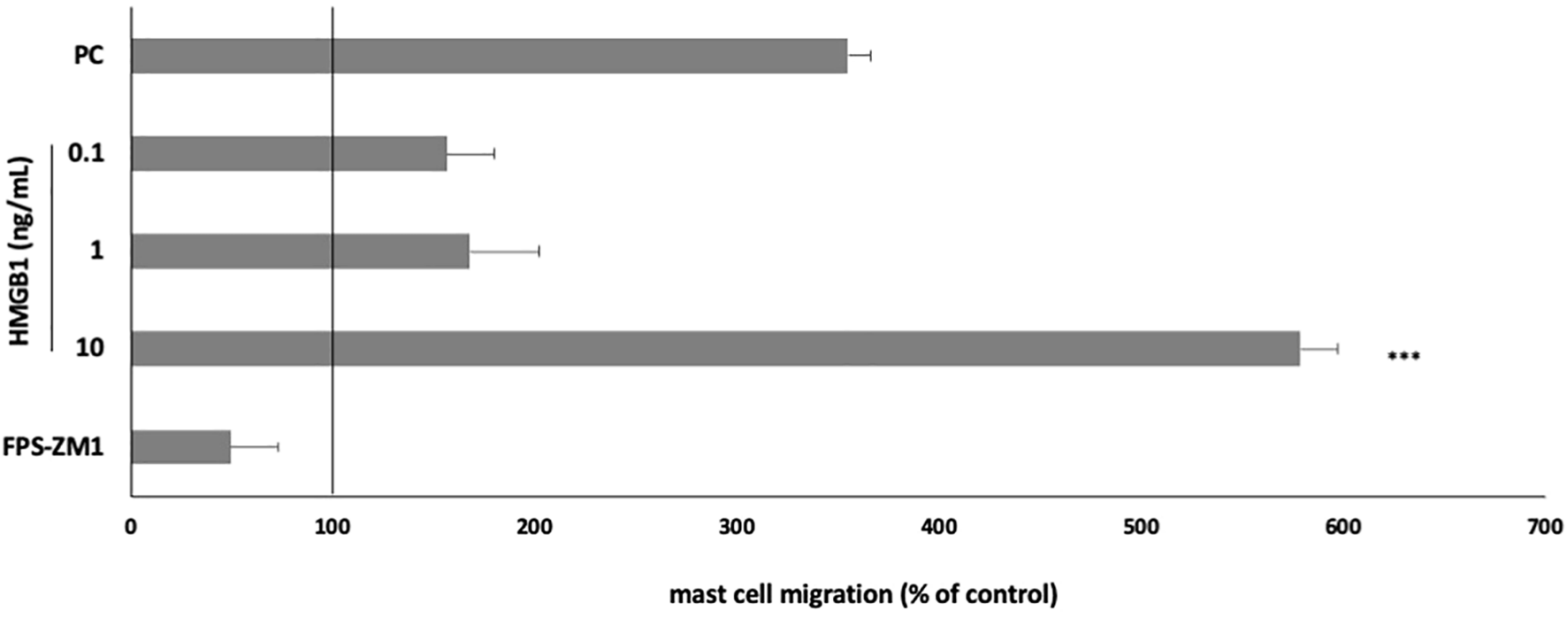
Effect of HMGB1 on MC migratory response. MCs were incubated with HMGB1 at 0.1–10 ng/mL, TNF at 0.05 pg/mL (positive control; PC), or medium alone (spontaneous MC migration) in a Boyden microchamber. RAGE antagonist FPS-ZM1 was used at 5 μM before the MCs incubation with 10 ng/mL HMGB1. Spontaneous migration served as a control and was referred to as 100%. Results are expressed as a percentage of spontaneous migration. Each point represents the mean ± SD of three independent experiments, and each experiment was performed in duplicate. Differences were considered significant at *P* < 0.05 and are labelled with an asterisk (*) on each graph (Student’s *t*-test). P*** < 0.001.

We also analyzed the potential of HMGB1 to initiate MC migration. Cells were incubated with HMGB1 for 3 h in a Boyden microchamber (at concentrations of 0.1–10 ng/mL) to determine its capability to induce MC migration. Results indicate that HMGB1 at a concentration of 10 ng/mL strongly influenced MC migration compared to spontaneous migration (578.89 ± 18.45% of control migration, *P* < 0.001) (**Figure 5**). One-way ANOVA also revealed statistically significant differences (*P* < 0.0001). Alarmin at the highest concentration acted as a much more potent cell chemoattractant than a well-known chemotactic factor, i.e., TNF. Pretreatment with RAGE antagonist completely inhibited the migratory MC response to HMGB1.

### 3.4 Involvement of signaling molecules in HMGB1-induced MC response

**Figure 6:**
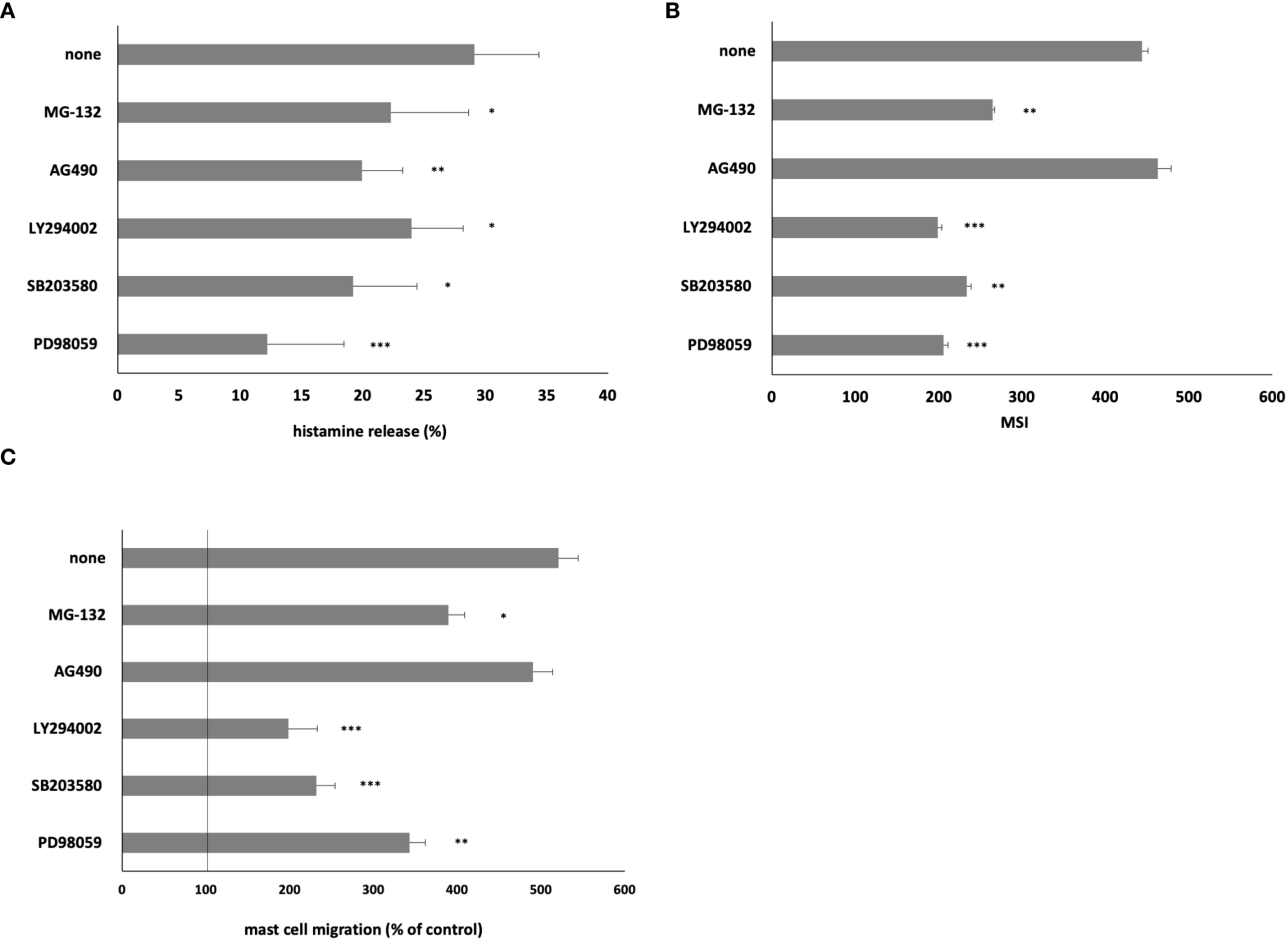
Effect of specific inhibitors of cell signaling molecules on MC **(A)** histamine release, **(B)** ROS generation, and **(C)** migration. MCs were preincubated with ERK1/2 inhibitor PD98059 at 50 μM, p38 inhibitor SB203580 at 10 μM, PI3K inhibitor LY294002 at 50 μM, JAK2 inhibitor AG490 at 10 μM, and NF-κB inhibitor MG-132 at 3 μM or medium alone prior to stimulation with HMGB1 at 10 ng/mL. Results are the mean ± SD of three independent experiments, and each experiment was performed in duplicate. Differences were considered significant at *P* < 0.05 and are labeled with an asterisk (*) on each graph (Student’s *t*-test), P* < 0.05, P** < 0.01, P*** < 0.001.

We conducted inhibitory experiments utilizing ERK1/2 inhibitor PD98059, p38 inhibitor SB203580, PI3K inhibitor LY294002, NF-κB inhibitor MG-132, and JAK2 inhibitor AG490 (**Figure 6**). Our documentation revealed that MC pretreatment with ERK1/2, p38, PI3K, and NF-κB inhibitors resulted in a markedly and statistically significant reduction in HMGB1-mediated histamine release (**Figure 6A**), ROS generation (**Figure 6B**), and migration (**Figure 6C**). Furthermore, pretreatment with JAK2 inhibitor AG490 elicited a statistically significant (*P* < 0.01) diminution in HMGB1-mediated histamine release (**Figure 6A**).

## 4 Discussion

Alarmins are constitutively stored in the cells of the innate immune system as components of granules, the cytoplasm, or the nucleus and can be regarded as primary responders to tissue damage. HMGB1, a non-histone nuclear protein, can function as an alarmin to promote inflammation (11). It can be released to alert neighboring cells about compromised homeostasis and can be internalized *via* the RAGE (32). As a prototypical DAMP, HMGB1 assumes a critical role in sterile inflammatory responses and significantly contributes to the development and progression of various non-communicable diseases, including atherosclerosis, autoimmune disorders, and chronic allergic inflammation. As discussed by Truong et al. (33), oxidized low-density lipoprotein enhances the secretion of DAMPs, including HMGB1, thereby initiating inflammation through binding to RAGE, TLR4, and CD36, which primes and activates the inflammasome in atherosclerosis. HMGB1 is reported to be upregulated in several autoimmune diseases, such as rheumatoid arthritis, systemic lupus erythematosus, type 1 diabetes mellitus, and autoimmune thyroid disease (34). The findings suggest by Zhu et al. (35) that HMGB1 and TLR4 are involved in the inflammatory response in allergic rhinitis through their interaction with various nasal interleukins, highlighting their potential role in the pathogenesis of this condition. The targeting of HMGB1 through neutralizing antibodies, inhibitors, or blocking its receptors (TLRs and RAGE) offers a promising avenue for asthma treatment, especially for patients with severe or steroid-resistant asthma (36).

A prominent sentinel of the immune system is the MC, which is characterized by a multitude of activities that uphold various physiological functions (17). MCs serve as pivotal coordinators in the immune response against invading microorganisms and are acknowledged as vital for modulating the inflammatory process (22, 24, 37, 38), which is further supported by our own observations (39–47). The relationship between HMGB1 and MC biology is poorly understood. Current evidence indicates a bidirectional interaction between HMGB1 and MCs, although existing data remain limited. Roy et al. (48) demonstrated that MC-derived chymase can degrade HMGB1, suggesting a potential regulatory mechanism through which MCs may influence HMGB1-mediated inflammation. Conversely, Gao et al. (49) reported that histamine can induce the translocation and secretion of HMGB1 from endothelial cells in a concentration- and time-dependent manner. Further corroborating this hypothesis, Wang et al. (50) found that MC degranulation in a urticaria model correlates with activation of the HMGB1–TLR4–NF-κB signaling pathway, an effect mitigated by paeoniflorin treatment. Similarly, Piao et al. (51) observed that glaucocalyxin A attenuates allergic inflammation *via* inhibition of the same HMGB1-dependent pathway. This report assessed the hypothesis that MCs serve as vital sensors of cell injury by responding to alarmins released from damaged cells. Our study aimed to evaluate the effect of HMGB1 on the fully mature MC phenotype, as measured by its influence on the expression of specific PRRs, including Dectin-1, Dectin-2, TLR2, NOD1, and RIG-I, as well as the pro-inflammatory activity of MCs. We indicated for the first time that HMGB1 could modulate to varying degrees the expression of some PRRs in MCs. We stated that MC stimulation with this alarmin enhanced Dectin-1 expression at mRNA and protein levels.

We also observed an increase in intracellular receptor expression, specifically NOD1 and RIG-I, as well as translocations of molecules from the nuclear region to the cell membrane following HMGB treatment. Notably, no significant changes were noted in Dectin-2 and TLR2 expression under the influence of alarmin. Previously, Qian et al. (52) reported similar findings, indicating that there were no changes in the expression levels of TLR2 and TLR4 in HMGB1-treated P815 cells. Dectin-1 is known to detect β-glucans, a critical component of fungal cell walls. NOD1 recognizes distinct motifs of PGN, which is an essential part of the bacterial cell wall, while RIG-I serves as a cytoplasmic sensor of viral RNA. Therefore, it can be inferred that the presence of this alarmin in the extracellular environment would enhance the MC’s capacity to recognize pathogen-associated molecular patterns (PAMPs). The observed translocation of intracellular receptors from the nuclear region to the cell membrane may suggest that MCs, under the influence of this protein, exhibit increased sensitivity to signals recognized by RIG-I.

Literature data suggest that HMGB1 directly influences immune cell populations. HMGB1 activates human peripheral blood monocytes and macrophages (53, 54) to produce a variety of cytokines such as IL-1, IL-6, TNF-α, and chemokines CCL3, CCL4, and CXCL8 (55, 56). The incubation of human neutrophils with HMGB1 results in the activation of the NF-κB pathway and the synthesis of various proinflammatory mediators (57). Furthermore, HMGB1 also activates dendritic cells (58–60). Treatment of myeloid immature dendritic cells with HMGB1 stimulates their maturation and induces the production of cytokines, including IL-1, IL-6, IL-12, TNF-α, and CXCL8. Additionally, HMGB1 enhances the capacity for migration in response to CCL21 and promotes the proliferation of allogeneic T lymphocytes. HMGB1 secreted by NK cells has been demonstrated to activate both B lymphocytes and dendritic cells, leading to the upregulation of inflammatory cytokines, including IL-6, IL-12, and TNF-α (61, 62). Therefore, HMGB1 serves as an activator for various leukocytes, including monocytes/macrophages, T cells, NK cells, and dendritic cells. This paper indicates that MC exposure to HMGB1 induces an increase in CCL3, CCL4, CCL5, IL-1β, IL-18, TGF-β, and TNF mRNA, as well as the release of CCL3, IL-1β, TNF, cysLTs, and histamine. HMGB1 also prompts the generation of significant amounts of ROS. Importantly, this protein acts as a potent chemoattractant for MCs.

The results presented herein are novel as they pertain to the activity of peritoneal MCs rather than cell lines. To date, Wang et al. (63) have found that HMGB1 can act as a potent inducer of the P815 MC line activation by enhancing Ca2^+^ influx and upregulating the CD117 expression. HMGB1 activates the PI3K/AKT/NF-κB and ERK/NF-κB signaling pathways, leading to an increase in the expression of TNF-α, IL-6, and tryptase. Furthermore, Qian et al. (52) demonstrated that the levels of TNF, IL-1β, and tryptase secreted into the supernatant significantly increased following the treatment of the P815 MCs with HMGB1. This alarmin also induced the accumulation of brain MCs in the hippocampal CA1 region.

Our results indicate that the alarmin under investigation may serve as a potent amplifier of MC activity in the context of inflammation. The findings indicating that HMGB1 activates MCs, leading to an increase in mRNA and protein levels of a panel of mediators, are of considerable significance. It is noteworthy that the mediators studied play a pivotal role in the regulation of inflammation, affecting vascular permeability and cell adhesion; thus, they are crucial mediators of leukocyte accumulation. Additionally, these mediators are vital for enhancing phagocyte activity and generating other pro-inflammatory mediators (64, 65). Among the cytokines that modulate the actions of macrophages, neutrophils, or endothelial cells, a predominant role is played by IL-1β, TNF, and the chemokine CCL3. Therefore, we consider our observations regarding the effect of HMGB1 on the generation of these mediators to be substantial. Among the inflammatory mediators implicated are the eicosanoids, such as cysLTs, and the axial MC player, histamine. Consequently, the increased synthesis of cysLTs and histamine facilitates both the early and chronic phases of inflammation. Furthermore, the observation that HMGB1 induces an increase in TGF-β mRNA expression is particularly noteworthy, as TGF-β is a pleiotropic cytokine possessing potent regulatory and anti-inflammatory properties. ROS function as both signaling molecules that regulate cell growth, mediate the adhesion of cells to one another, and mediate inflammation.

Furthermore, we have demonstrated that HMGB1, at low concentrations, significantly facilitates MC migration. This observation is indeed pioneering. Consequently, it can be inferred that the presence of HMGB1 in the extracellular environment at the locus of cell or tissue damage may stimulate MC infiltration and their aggregation at the site of injury. Given that MCs are recognized for their essential role in wound healing and tissue repair, the presence of a considerable number of MCs within damaged tissue may be critical for the progression of repair processes.

The activation of cells by HMGB1 is partially dependent on the RAGE (32, 66). RAGE is a component of the immunoglobulin superfamily of cell surface molecules and is essential for HMGB1 to execute its inflammatory and immunoenhancing effects. Our findings demonstrated that the RAGE antagonist significantly diminished the CCL3/IL-1β/TNF/ROS/histamine generation and migratory response, thereby confirming the role of this receptor in the cellular response under the influence of HMGB1. We present compelling evidence indicating that various intracellular signaling pathways are involved in the HMGB1-mediated activation of MCs. Pretreatment with specific inhibitors targeting ERK1/2, p38 MAPK, PI3K, and NF-κB significantly reduced HMGB1-induced histamine release, ROS production, and cell migration, thereby underscoring the critical role of these pathways in mediating pro-inflammatory responses. Notably, the inhibition of JAK2 with AG490 also led to a statistically significant decrease in HMGB1-induced histamine release, thereby emphasizing the contribution of JAK/STAT signaling pathway in this process. These findings suggest that HMGB1 activates MCs through a complex network of signaling cascades, and that pharmacological intervention targeting these pathways may provide promising therapeutic strategies for managing HMGB1-associated inflammatory diseases.

It is widely acknowledged that HMGB1 can be passively released into the extracellular environment from damaged or necrotic cells in response to injuries and infections, or actively from stimulated immune cells. There is evidence that, in numerous inflammatory and infectious conditions, the levels of extracellular HMGB1 are elevated. Consequently, HMGB1 is currently regarded as both a crucial alarmin and a proinflammatory cytokine. In conclusion, accumulating evidence underscores the central role of HMGB1 in the regulation of inflammatory and allergic responses across various organ systems, particularly in diseases where MCs play a crucial role. In asthma, HMGB1 functions as a pro-inflammatory alarmin and therapeutic target, interacting closely with MC-mediated pathways that drive airway inflammation and hyperresponsiveness (67, 68). Similarly, in inflammatory skin diseases such as atopic dermatitis and allergic contact dermatitis, HMGB1 contributes to disease progression not only through direct immune activation and chromatin remodeling but also *via* modulation of MC degranulation and mediator release (69–71). Moreover, chronic inflammation sustained by HMGB1 involves MC-derived factors that promote tissue fibrosis and remodeling, as evidenced in Sjögren’s syndrome-related sialadenitis (72, 73). These findings highlight the critical interplay between HMGB1 and MCs in amplifying and maintaining inflammatory responses, supporting the potential of targeting this axis as a therapeutic strategy in MC-associated immune disorders. This report demonstrates that HMGB1 stimulates MCs to produce pro-inflammatory and immunoregulatory mediators, upregulates the expression of specific PRRs, and acts as a chemoattractant for MCs. We support the concept that HMGB1 is a key endogenous factor that promotes and amplifies MC activation, thereby contributing significantly to the propagation of inflammatory responses.

## Data Availability

The original contributions presented in the study are included in the article/**Supplementary Material**. Further inquiries can be directed to the corresponding author.
