# Supplementary material for: HMGB1-dependent signaling in the regulation of mast cell activity during inflammation
Source: Front Immunol. 2025 Oct 3;16:1643427. doi: 10.3389/fimmu.2025.1643427 (PMC12531030; doi:10.3389/fimmu.2025.1643427)
Supplement: Supplementary file 1 [file DataSheet1.zip › Table 3.DOCX]

**Supplementary Table 3.** P-values for all pairwise comparisons of migratory response, calculated using ANOVA followed by Tukey’s post hoc test. **NS refers to non-stimulated cells.** Comparisons that did not reach statistical significance are labeled as “ns” (not significant).

| **Figure** | **Post-hoc comparison** | ***P*-value** |
| --- | --- | --- |
| 5 | NS *vs.* 0.1 ng/mL HMGB1 | *P* < 0.001 |
|  | NS *vs.* 1 ng/mL HMGB1 | *P* < 0.0001 |
|  | NS *vs.* 10 ng/mL HMGB1 | ns |
|  | 0.1 ng/mL HMGB1 *vs.* 1 ng/mL HMGB1 | ns |
|  | 0.1 ng/mL HMGB1 *vs.* 10 ng/mL HMGB1 | ns |
|  | 1 ng/mL HMGB1 *vs.* 10 ng/mL HMGB1 | ns |
