# Supplementary material for: HMGB1-dependent signaling in the regulation of mast cell activity during inflammation
Source: Front Immunol. 2025 Oct 3;16:1643427. doi: 10.3389/fimmu.2025.1643427 (PMC12531030; doi:10.3389/fimmu.2025.1643427)
Supplement: Supplementary file 1 [file DataSheet1.zip › Table 1.DOCX]

**Supplementary Table 1.** *P*-values obtained from one-way ANOVA followed by Tukey’s post hoc test for all pairwise comparisons of receptor expression levels. **NS refers to non-stimulated cells.** Comparisons not reaching statistical significance are indicated as “ns” (not significant).

| **Figure** | **Receptor** | **Post-hoc comparison** | ***P*-value** |
| --- | --- | --- | --- |
| 1C | Dectin-1 | NS vs. 1 h | ns |
|  |  | NS vs. 3 h | ns |
|  |  | 1 h vs. 3h | ns |
|  | Dectin-2 | NS vs. 1 h | *P* < 0.0001 |
|  |  | NS vs. 3 h | *P* < 0.0001 |
|  |  | 1 h vs. 3h | ns |
|  | TLR2 | NS vs. 1 h | ns |
|  |  | NS vs. 3 h | ns |
|  |  | 1 h vs. 3h | ns |
| 2C | NOD1 | NS vs. 1 h | *P* < 0.0001 |
|  |  | NS vs. 3 h | *P* < 0.0001 |
|  |  | 1 h vs. 3h | *P* < 0.0001 |
|  | RIG-I | NS vs. 1 h | *P* < 0.05 |
|  |  | NS vs. 3 h | ns |
|  |  | 1 h vs. 3h | *P* < 0.0001 |
